# An Extract of *Artemisia argyi* Leaves Rich in Organic Acids and Flavonoids Promotes Growth in BALB/c Mice by Regulating Intestinal Flora

**DOI:** 10.3390/ani12121519

**Published:** 2022-06-10

**Authors:** Qianbo Ma, Dejin Tan, Xiaoxiao Gong, Huiming Ji, Kexin Wang, Qian Lei, Guoqi Zhao

**Affiliations:** College of Animal Science and Technology, Yangzhou University, Yangzhou 225009, China; mqb1601555436@163.com (Q.M.); tan1837305368@163.com (D.T.); 18762329160@163.com (X.G.); jhm_1996@163.com (H.J.); awkx5527@163.com (K.W.); lq92779277@163.com (Q.L.)

**Keywords:** *Artemisia argyi*, extract, mouse model, intestinal flora

## Abstract

**Simple Summary:**

With the development of the economy, people are paying more attention to their health. Regular eating habits and quality ingredients are becoming increasingly popular. As an important human food source, the safety of animal products has received more attention. In China, there is a long history of research on Chinese herbal medicine. Many Chinese herbal medicines have been used in animal husbandry because of their naturally low toxicity and various active functions. *Artemisia argyi* (*A. argyi*) is a Chinese herbal medicine with a long history of use. It has antibacterial, anti-inflammatory and blood activating functions. In this study, *A. argyi* leaves extract was investigated to determine if it has positive regulatory effects on animal growth in order to develop its potential as a plant-derived feed additive.

**Abstract:**

In the context of global restrictions on the use of antibiotics, there has been increased research on natural plant-based ingredients as additives. It has been proved that many natural active ingredients contained in plants have positive effects on animal growth regulation. *Artemisia argyi* (*A. argyi*) is a traditional Chinese herbal medicine, and its extracts have been reported to have a variety of biological activities. Therefore, in order to explore the potential of the active extract of *Artemisia argyi* leaves (ALE) as a plant source additive, mice were fed with ALE at different concentrations for 60 days. Finally, the effects of ALE were evaluated by the growth indexes, blood indexes, and intestinal microflora changes of the mice. It was found that a medium concentration of ALE (150 mg/kg) could promote growth, and especially improved the feed efficiency of the mice. However, high concentrations of ALE (300 mg/kg) had some negative effects on the growth of mice, especially liver damage, which significantly increased AST and ALT levels in the blood. Therefore, the 150 mg/kg ALE treatment group was selected for 16S rDNA analysis. It was found that ALE could play a positive role by regulating the proportion of *Bacteroidetes* and *Firmicutes* in the intestinal tract. In particular, it can significantly up-regulate the quantities of *Akkermansia* and *Bifidobacterium*. These results suggest that ALE at appropriate concentrations can positively regulate animal growth.

## 1. Introduction

Livestock products are an important part of human food. And the safety of livestock products has increasingly received attention. For this reason, the use of substances other than herbal additives to regulate livestock growth has been restricted in China [1]. An abundant variety of Chinese herbs exists and their sources are extensive. They are very safe as they contain no artificial additives and have a long history of use in China. At present, the utilization of Chinese herbal materials in the field of animal husbandry, mainly occurs through direct feeding or extraction of active ingredients. For example, adding ginger to a broiler diet could increase the quality of broiler [2]. In ruminant feeding, the extract also showed a positive promotion effect. It was found that milk yield and milk composition could be improved by adding *Coleus amboinicus* L. leaves extract to Ettawa crossbred goat diets [3].

*Artemisia argyi* (*A. argyi*), a kind of traditional Chinese herbal medicine, has been proven to be rich in natural active substances [4]. Volatile oil is the main active substance in *A. argyi*, which has been shown to have antioxidant [5], antibacterial [6], and anti-inflammatory [7] effects. Studies have also found that *A. argyi* contains flavonoids, organic acids, polysaccharides, and other substances. There have been many studies on the use of *A. argyi* in livestock feeding. In rabbit feeding, it was found that adding *A. argyi* can improve intestinal immunity [8]. Studies on the aqueous extract of *A. argyi* as additive have also been reported. It was found that adding 1000 mg/kg *A. argyi* aqueous extract to broilers can improve the antioxidant capacity of the small intestine [9]. From the above studies, it is not difficult to see that *A. argyi* can regulate and improve the intestinal function of animals, but there are few studies on the specific active components.

The intestine is considered to be the most important and complex ecosystem [10]. The intestinal microflora are members of this complex ecosystem, with more than 400 species of bacteria already identified [11]. Recent studies have found that intestinal microflora dysfunction can lead to intestinal inflammation and further lead to a range of diseases [12,13]. Some studies have also found that the metabolites of intestinal flora are involved in the key pathways that promote inflammation, fibrosis, and genotoxicity [14,15]. Therefore, changes in intestinal flora can reflect changes in the growth state of the body, which means that it can be studied as a marker of changes in the body.

In the previous study, we obtained an extraction of *A. argyi* leaves (ALE) and found it was mainly composed of flavonoids and organic acids by using high performance liquid chromatography tandem mass spectrometry (HPLC-MS) identification [16]. Therefore, in order to further explore the effects of ALE on animal growth, we selected mice as a model to discover the mechanism of ALE action in vivo through a series of intestinal flora changes.

## 2. Materials and Methods

### 2.1. Preparation of A. argyi Leaves Extract

**Figure 1:**
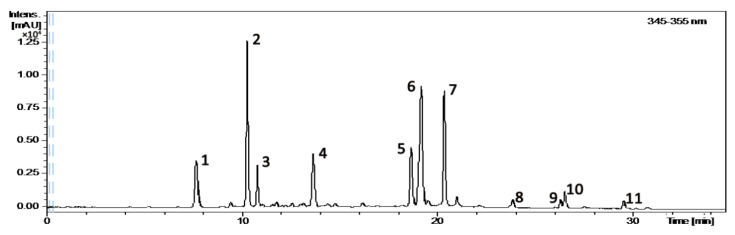
The HPLC chromatogram at 350 nm of ALE.

*A. argyi* were harvested in Suqian, Jiangsu, China. The leaves of *A. argyi* were placed in an open area away from the sun to dry naturally. Liposoluble substances and pigment of the *A. argyi* leaves were removed using petroleum ether with a boiling range of 60–90 °C. Then, *A. argyi* leaves were extracted with 95% ethanol after removal of the petroleum ether. Next, the combined and concentrated extract was extracted again with water three times, and the system was separated with a high-speed centrifuge to obtain the supernatant. Finally, the *A. argyi* leaves extract’s (ALE) dry powder was prepared by freeze-drying. The main components were separated and identified by HPLC-MS. The chromatogram of the ALE is shown in Figure 1 and the identification results of each component are shown in Table 1.

### 2.2. Animals

Forty SPF BALB/c female mice aged 6–8 weeks (16.5 ± 0.5 g) were purchased from the Animal Experiment Center of Yangzhou University (Yangzhou, China). The purchased mice were divided into 4 groups with 10 mice in each group. All experiments were performed with approval of the protocols by the guidelines of the Institutional Animal Care and Use Committee (IACUC) of Yangzhou University under permit number SYXK(Su) IACUC 2012-0029. One week before the official experiment, the mice were transferred to the experimental environment for adaptive feeding, during which they were free to eat and drink. Then, the ALE evaluation experiment lasted 60 days. Irradiation sterilized maintained feed for laboratory mice (Xietong Biotechnology, Yangzhou, China) was selected as the feed, and the nutritional composition of the feed was shown in Table 2.

### 2.3. Experimental Designs

ALE was directly injected into the stomach of mice with a special syringe. In this way, three supplemental concentrations of ALE were determined through pre-experiment: high concentration group (H, 300 mg/kg), medium concentration group (M, 150 mg/kg), and low concentration group (L, 75 mg/kg), with an equal volume (50 μL) of normal saline was used as a control check (CK). In the formal experiment, the mice were perfused with the ALE once every morning, and the rest of the time were free to eat and drink. The experiment lasted 60 days.

### 2.4. Growth Indexes of Mice

#### 2.4.1. Average Body Weight of Mice

To evaluate the relationship between feeding days and average body weight, the weight of each mouse was recorded before the formal experiment. The total body weight of each group before perfusion was recorded on days 10, 20, 30, 40, 50, and 60. The average body weight of each group was then calculated.

#### 2.4.2. Average Growth Rate of Mice

To evaluate the average growth rate of mice, the ratio of the weight gain of each group of mice every ten days to the initial total body weight was calculated.

#### 2.4.3. Feed Conversion Rate of Mice

To evaluate the effects of ALE on feed intake in mice, the feed conversion rate was evaluated by calculating the ratio of the total feed intake to the total body weight gain of mice in each group within 60 days.

### 2.5. Development Indexes of Mice

At the end of 60 days of perfusion feeding, water and food were cut off for 24 h. After blood was taken, the mice were euthanized. The heart, liver, and spleen were weighed and dissected to obtain the weight, and the relative weight was calculated.

### 2.6. Blood Indexes of Mice

After 60 days of perfusion, 2 mL of blood was collected from the mice orbit. 1 mL was placed in ordinary centrifugal tubes for 1.5–2.0 h and centrifuged at 4 at 3000 r/min, and after 15 min, the serum was absorbed into a new centrifuge tube and stored in a cryogenic refrigerator at −80 °C for serological indexes detection. The serum of mice was determined by automatic serum biochemical analyzer (BS-280, Mindray, Yangzhou, China). The other 1 mL was placed in a heparin sodium tube for hematological tests. Automatic animal hematology analyzer (BC-2800Vet, Mindray, Yangzhou, China) was used to measure blood indexes.

### 2.7. 16s rDNA Sequencing and Bioinformatics Analysis

#### 2.7.1. Collection of Feces Samples from Mice

On the 60th day of feeding, fresh feces of mice were collected, and about 6 feces (more than 50 mg) were collected from each mouse. The feces of mice were placed in a fingerless tube without enzyme and stored at −80 °C for further treatment after rapid cooling with liquid nitrogen.

#### 2.7.2. DNA Extraction and HiSeq Platform Sequencing

The TIANamp Stool DNA Kit for faeces (TIANGEN, Shanghai, China) was used to obtain metagenomic DNA from mouse faeces according to the manufacturer’s instructions. 30 ng of qualified genomic DNA samples and corresponding fusion primers were taken to configure PCR reaction system, and PCR reaction parameters were set for PCR amplification. The primers were as follows: 515F (5′-GTGCCAGCMGCCGCGGTAA-3′) and 806R (5′-GGACTACHVGGGTWTCTAAT-3′). Agencourt AMPure XP magnetic beads (Beckman Coulter, Brea, CA, USA) were used to purify the PCR products and dissolve in the elution buffer to complete the construction of the library. Agilent 2100 Bioanalyzer (Agilent Technologies, Santa Clara, CA, USA) was used to detect the fragment range and concentration of the library. HiSeq platform was selected for sequencing of qualified libraries according to the inserted fragment size. The HiSeq platform was performed by BGI Biological Technology Co., Ltd. (Wuhan, China).

#### 2.7.3. Data Filtering

In order to obtain clean data, the sequencing data were processed as follows. Firstly, the sequence was screened preliminarily according to the window method for removing low quality. Secondly, by setting the allowable sequence mismatch to 3 bp, the reads contaminated by the joint were removed. Thirdly, reads containing nitrogen were removed. Fourthly, low-complexity reads were removed.

#### 2.7.4. Tags Connection

Sequences were spliced using Usearch method and FLASH (Fast Length Adjustment of Short reads, V1.2.11).

#### 2.7.5. Operational Taxonomic Unit (OTU) Cluster Analysis

UPARSE was used for clustering with 97% similarity to obtain OTU representative sequences. UCHIME (V4.2.40) was used to remove chimeras generated by PCR amplification from OTU representative sequences. The taxonomy of each 16s rDNA gene sequence was analyzed by the Ribosomal Database Projecct (RDP) Classifer (V1.9.1) against the Greengene database with a confidence threshold of 60%. Usearch_global method was used to compare all Tags back to the OTU representative sequence, and the OTU abundance statistics table of each sample was obtained.

#### 2.7.6. α-Diversity Analysis

Mothur software v1.28.0 (The University of Michigan, Ann Arbor, MI, USA) and R language (University of Auckland, Auckland, New Zealand) were used to analyze the α-diversity of intestinal flora. The diversity analysis includes Sob, Chao, Ace, Shannon, and Simpsom indices.

#### 2.7.7. LDA Effect Size (LEfSe) Analysis

LEfSe analysis is for discovering high dimensional biomarkers and revealing genomic features. It was used to distinguish genomic features between two or more microbial communities and to emphasize statistical significance and biological correlation. LEfSe analysis results were provided by BGI Biological Technology Co., Ltd (Wuhan, China).

### 2.8. Statistical Analyses

All the pre-processed data were sorted and calculated by Excel, and then analyzed by SPSS 22.0 software (IBM, Armok, NY, USA). The growth and blood indexes of mice were analyzed by one-way ANOVA. Duncan method was used for comparison. T test was used for 16s rDNA sequencing and bioinformatics analysis. All of the test results were expressed as mean ± SEM. *p* < 0.05 was considered statistically significant. In addition to 16S rDNA analysis, images were made by Graphpad Prism 6.0 software (GraphPad Software, San Diego, CA, USA). Analysis data of 16S rDNA were provided by BGI Biological Technology Co., Ltd (Wuhan, China).

## 3. Results

### 3.1. Effects of ALE on Growth of Mice

#### 3.1.1. Changes in Body Weight

**Figure 2:**
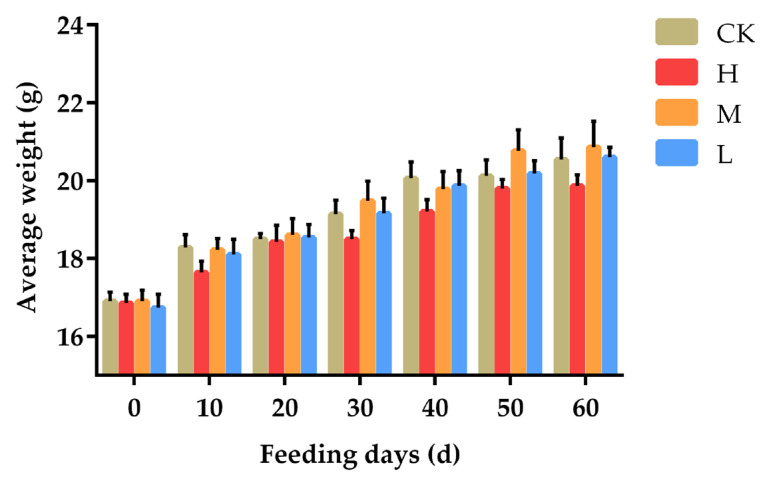
Effects of ALE on body weight of mice. Note: CK: 50 μL 0.9% NaCl; H: 50 μL 300 mg/kg ALE; M: 50 μL 150 mg/kg ALE; L: 50 μL 75 mg/kg.

The changes in body weight of mice were evaluated by two methods. It can be seen from Figure 2 that with the increase of feeding days, the body weight of mice in each group kept rising. In the same feeding days, there was no significant difference in body weight between ALE groups and the CK group, but the H group showed a decreasing trend at all time points, especially on the 10th day (*p* = 0.191) and the 40th day (*p* = 0.147). The M group showed a tendency to outperform the control group at the 50th day and the 60th day. At the end of the 60th day, the highest average weight of mice in M group was 20.86 g, and the lowest average weight of mice in H group was 19.86 g.

**Figure 3:**
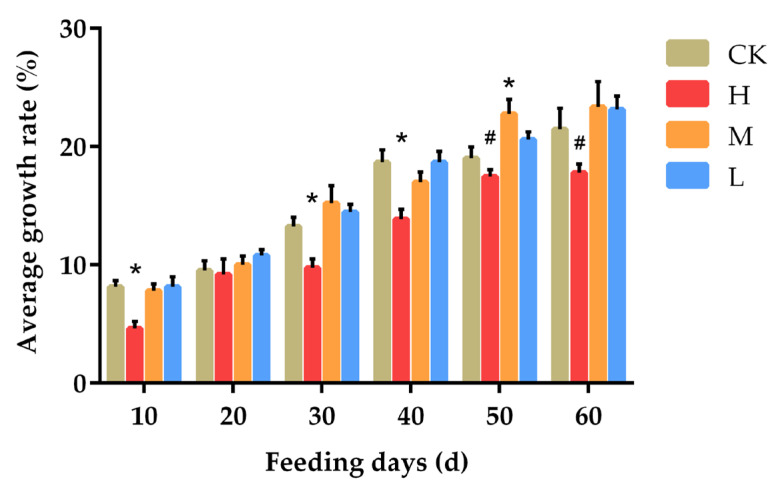
Effects of ALE on body weight growth in mice. Note: CK: 50 μL 0.9% NaCl; H: 50 μL 300 mg/kg ALE; M: 50 μL 150 mg/kg ALE; L: 50 μL 75 mg/kg. * *p* < 0.05 vs. CK group; # *p* < 0.05 vs. M group.

In order to more directly reflect the rate of change of mouse body weight, Figure 3 was obtained by calculating the rate of increase body weight of mice. It can be seen in Figure 3 that the increase of body weight of the mice was more obvious, and the growth rate of the H group was significantly lower than that of the control group on days 10, 30, and 40 (*p* < 0.05). On the 50th day, the growth rate of the M group was significantly higher than that of the CK group (*p* < 0.05), while the H group showed a downward trend compared with the CK group, but the difference was not significant (*p* = 0.229). On the 60th day, compared with the control group, the H group also showed a downward trend (*p* = 0.114), but was significantly lower than the M group (*p* < 0.05).

#### 3.1.2. Changes in Feed Coversion Rate

**Figure 4:**
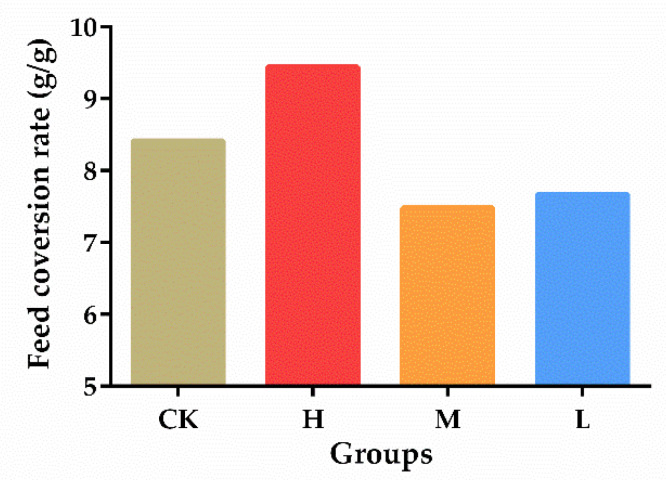
Effects of ALE on feed conversion rate in mice. Note: CK: 50 μL 0.9% NaCl; H: 50 μL 300 mg/kg ALE; M: 50 μL 150 mg/kg ALE; L: 50 μL 75 mg/kg.

It can be seen from Figure 4 that there were differences in feed conversion rate among different groups. Compared with the other groups, the highest amount of feed consumed by mice in the H group for gaining 1 g body weight was about 9.44 g. The M group consumed only 7.48 g feed for each 1 g of body weight gained.

### 3.2. Effects of ALE on Development in Mice

The mice were weighed before being killed, and the hearts, livers, and spleens isolated from dissected mice were also weighed. It can be seen from Table 3 that the accurate weight of mice after one day of food and water deprivation, the average weight of mice in the M group was higher than that in the CK group, while the average weight of mice in the H group was the lowest, but the difference was not significant. There was no significant difference in the quality of other organs except the heart. Compared with the CK group, the heart quality of the M group was significantly higher than that of the CK group and the high-dose group (*p* = 0.007), and the proportion of heart to body weight was significantly higher than that of the CK group (*p* = 0.022). The spleen quality was the lowest in the H group and the highest in the M group, but the difference was not significant (*p* = 0.075).

### 3.3. Effects of ALE on Blood Indexes in Mice

#### 3.3.1. Effects of ALE on Blood Routine in Mice

As can be seen from Table 4, white blood cell count (WBC) and granulocyte count (GRAN) in the H group were significantly higher than those in the CK group (*p* < 0.05). The number of monocyte count (MON) decreased significantly with the addition of ALE (*p* < 0.05). The number of GRAN in M group was significantly lower than that in control group (*p* < 0.05). There were no significant differences in all indexes except the number of MON between the L group and the CK group (*p* > 0.05). There was no significant difference in lymphocyte count (LYM), red blood cell count (RBC), hemoglobin concentration (HGB), hematocrit (HCT), and platelets (PLT) (*p* > 0.05).

#### 3.3.2. Effects of ALE on Serum Biochemical Indices in Mice

According to the results in Table 5, the addition of high concentration ALE significantly increased the activity of alanine transaminase (ALT) and aspartate aminotransferase (AST) (*p* < 0.05). The addition of medium and low concentration of ALE had no significant effect on the two enzymes (*p* > 0.05). ALE supplementation had no significant effect on the contents of total protein (TP), albumin (ALB), and globulin (GLOB).

### 3.4. Effects of ALE on Intestinal Flora

Combined with the above experimental results, the feces of the M group were selected as the treatment group (T), and the CK group was selected as the control group (CON) for 16s rDNA analysis.

#### 3.4.1. OTU Statistics

**Figure 5:**
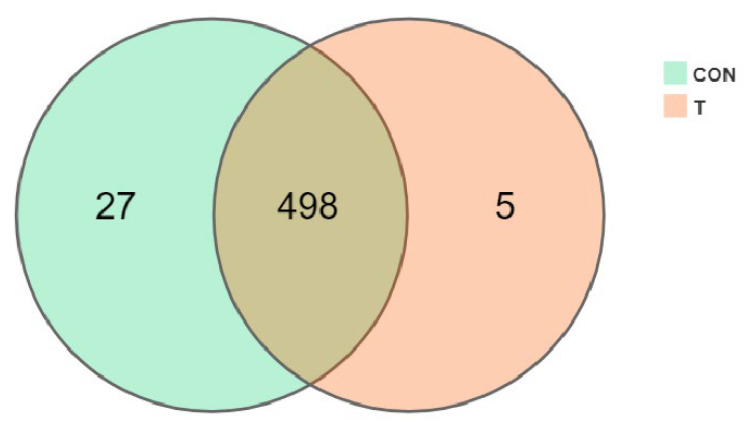
Influence of ALE on OTU.

Figure 5 shows the processing results of OTU after pre-processing screening. It can be seen that the OTU number of the CON group and the T group was 525 and 503 respectively, and the same OTU number was 498.

**Figure 6:**
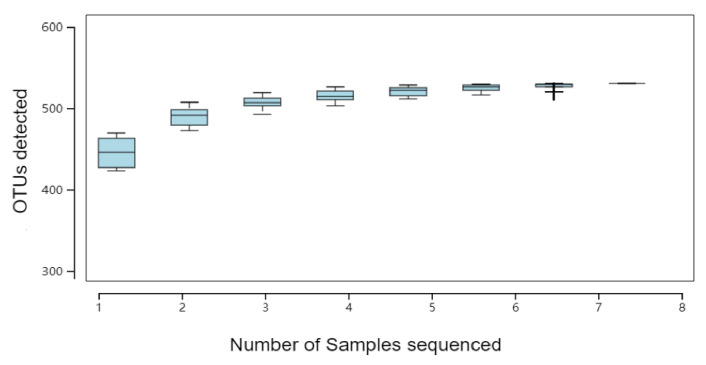
Species accumulation curve.

The cumulative curve of species reflects the effect of sampling numbers of species diversity. The abscissa represents the number of samples and the ordinate represents the number of species detected. As can be seen from Figure 6, the upward trend at the end of the curve tends to be gentle as the number of samples increases.

**Figure 7:**
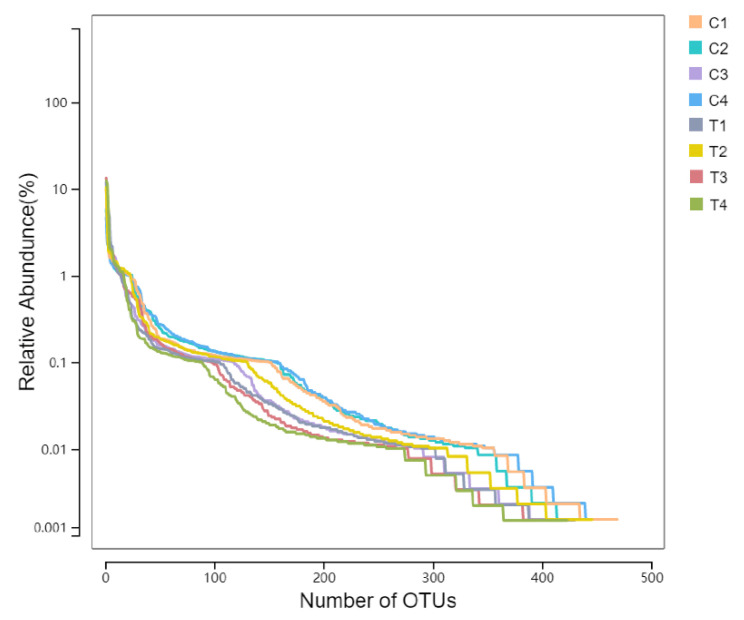
RANK curve of species.

Figure 7 showed the degree of species richness and evenness of species composition. The abscissa is the OTU grade of the sample, and the ordinate is the relative abundance of OTU. It can be seen that the relative abundance of species was mainly concentrated in 0.1–1% and 0.01–0.1%. The curves of all samples were wide, and the curves of all samples were flat in the range of 0.01–0.1%.

#### 3.4.2. Analysis of α Diversity of Intestinal Flora

**Figure 8:**
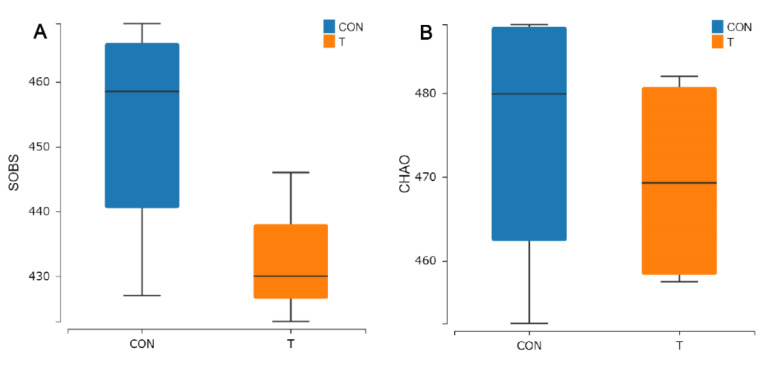

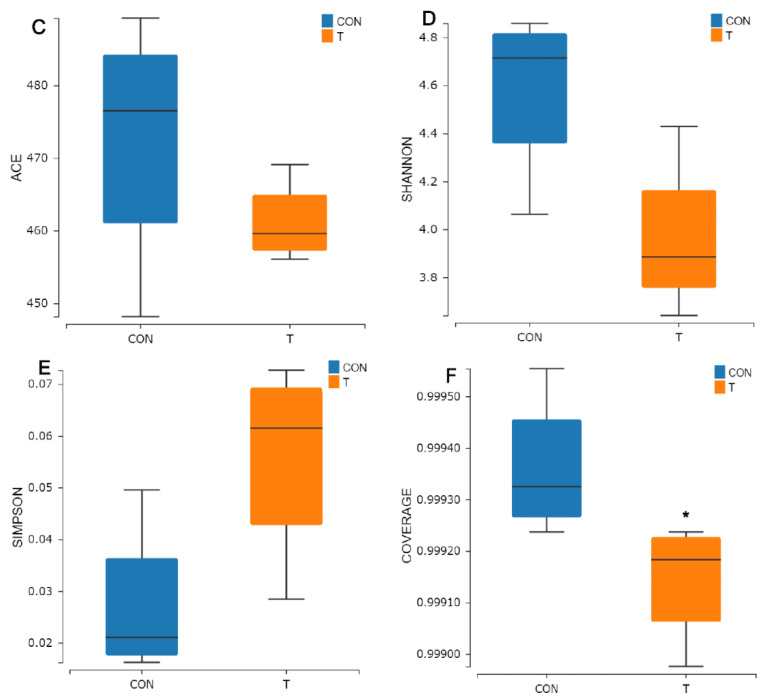
Comparison of ALE on α diversity indexes of intestinal microbial in mice. (**A**) Bacterial community richness index (SOBS); (**B**) Bacterial community richness index (CHAO); (**C**) Bacterial community richness index (ACE); (**D**) Bacterial community diversity index (SHANNON); (**E**) Bacterial community diversity index (SIMPSON); (**F**) Bacterial community diversity index (COVERAGE). * indicates significant difference between groups (*p* < 0.05), and no * indicates no significant difference (*p* > 0.05).

Figure 8A–F were box graphs of α diversity in the CON and T groups. Wilcox Test was used for comparison between the two groups. Compared with the CON group, Sobs, Chao, Ace, Shannon, and Simpson of mice in the T group showed no statistical difference Figure 8A–E, but Sobs (*p* = 0.191), Ace (*p* = 0.342), and Shannon (*p* = 0.057) showed a decreasing trend potential, while Simpson showed an opposite trend (*p* = 0.057). As can be seen from Figure 8F, compared with the CON group, Coverage of the T group decreased significantly (*p* = 0.042).

#### 3.4.3. Metastats Analysis of Intestinal Microflora in Mice

**Figure 9:**
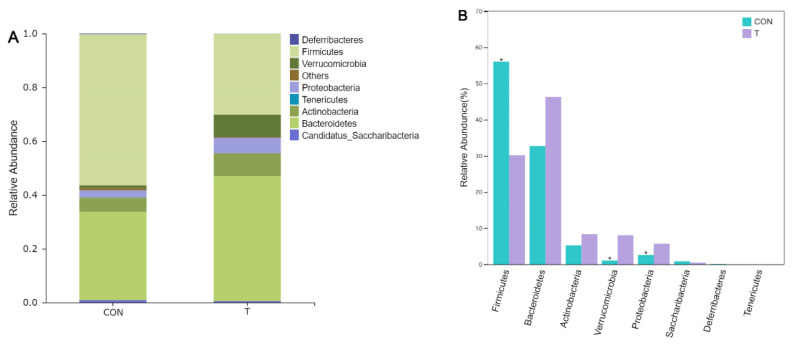
(**A**) Comparison of intestinal bacteria species distribution in mice at phylum level; (**B**) Comparison of intestinal bacteria species classification in mice at phylum level. * indicates significant difference between groups (*p* < 0.05), and no * indicates no significant difference (*p* > 0.05).

In order to investigate differences in microbial community abundance among groups, Metastats was used to analyze the differences in bacterial taxonomic composition between the CON group and the T group. As can be seen from Figure 9A, at the phylum level, the intestinal flora of the CON group and the T group mainly contained two phylum level bacteria, *Firmicutes* and *Bacteroidetes*. Combined with Figure 9B, the abundance of *Firmicutes* in the T group was significantly decreased compared with that in the CON group (*p* < 0.05), and the abundance of *Verrucomicrobia* and *Proteobacteria* increased significantly (*p* < 0.05). The abundance of *Bacteroidetes* in the T group was higher than that in the CON group, but there was no significant difference (*p* = 0.194). The abundance of *Actinobacteria* was also high, but there was no significant difference between the two groups (*p* > 0.05).

**Figure 10:**
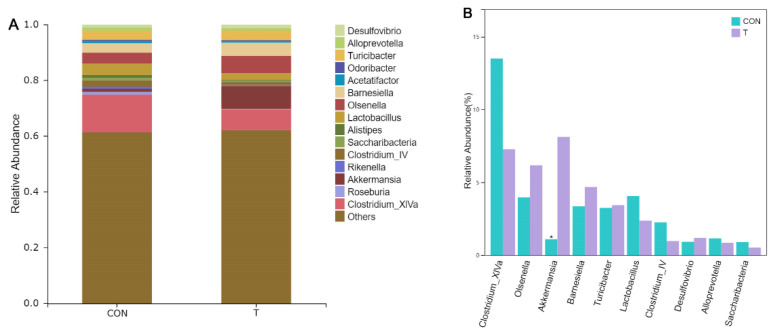
(**A**) Comparison of intestinal bacteria species distribution in mice at genus level; (**B**) Comparison of intestinal bacteria species classification in mice at genus level. * indicates significant difference between groups (*p* < 0.05), and no * indicates no significant difference (*p* > 0.05).

In order to further explore the differences in the composition of bacteria, further analysis was conducted at the genus level with the same analytical method at the phylum level. Combined with Figure 10A,B, it can be found that the abundance of *Akkermansia* in the T group increased significantly compared with the CON group (*p* < 0.05), belonging to *Verrucomicrobia*, and there was no significant difference among the other microflora at genus level. Figure 10B showed that the *Clostridum IV* and *XIVa* were the main genus in the flora. Although the addition of extracts has no significant effect on the abundance of them compared with the control group, the abundance decreases numerically. *Barnesiella* and *Olsenella* were also the most abundant at the genus level, and their values showed an upward trend with the treatment of ALE.

#### 3.4.4. LEfSe Analysis of Intestinal Flora in Mice

**Figure 11:**
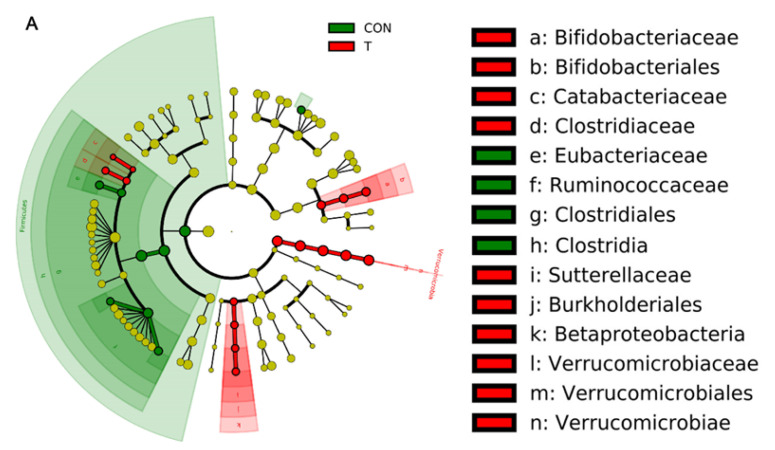

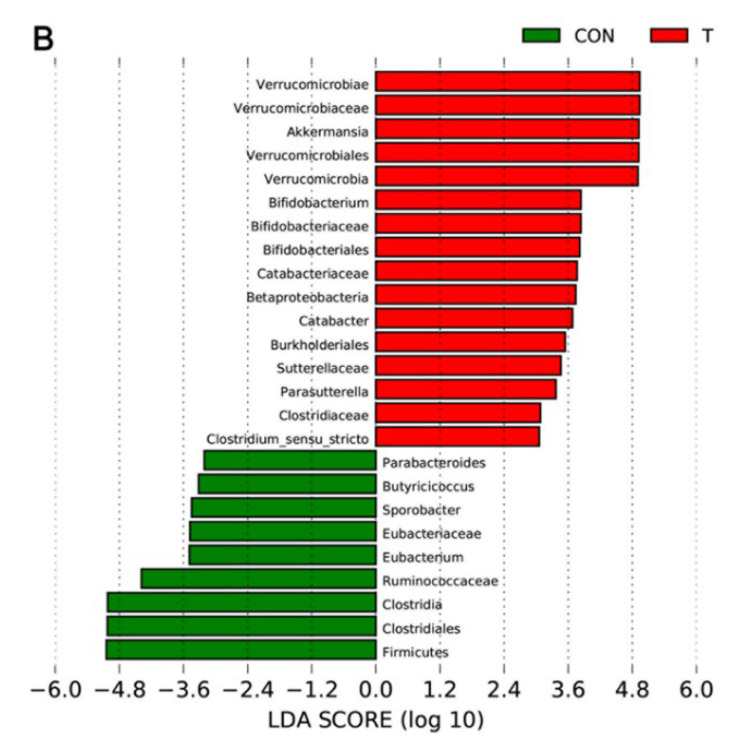
(**A**) Evolutionary branching of intestinal flora of mice in groups of control and treatment; (**B**) Comparison of linear discriminant analysis (LDA) of intestinal flora of mice in groups of control and treatment. In (**A**), the circles radiating from the inside out represent the classification level from phylum to genus (or species). Each small circle at a different classification level represents a classification at that level. Each small circle at a different classification level represents a classification at that level. The red nodes represent the microbiome that plays an important role in the treatment group, and the green nodes represent the microbiome that plays an important role in the control group. Species with no significant difference are yellow nodes. (**B**) shows species with differences in LDA scores above the set point (2.0), i.e., statistically significant differences.

LEfSe analysis can be used to further evaluate the effect of ALE on the composition of intestinal flora in mice. By identifying the specific altered bacterial phenotypes (LDA score > 2.0), Figure 11A,B were obtained. Figure 11A was the LEfSe cladogram. Nodes of different colors represent the microorganisms that play an important role in the grouping represented by this color. Meanwhile, from inside to outside, each circle represents the species at phylum, class, order, family, and genus level in turn. Based on this, compared with the CON group, there were three different microbiota in the added ALE group, which were *Bifidobacteriaceae*, *Bifidobacteriales* (a, b), *Verrucomicrobiaceae*, *Verrucomicrobiales*, *Verrucomicrobiae* (l, m, n) and *Sutterellaceae*, *Burkholderiales*, and *Betaproteobacteria* (i, j, k). Meanwhile, *Clostridiales*, *Clostridia* (g, h) played an important role in the CON group.

In Figure 11B, LDA score was selected to more intuitively show the composition of different microflora. Compared with the CON group, a total of 25 bacterial groups were significantly changed in the T group, including 9 down-regulated and 16 up-regulated bacteria groups. At phylum classification level, the ALE significantly down-regulated *Firmicutes* and up-regulated *Verrucomicrobia* (*p* < 0.05). From class classification level, further identified the action of the two classes, *Clostridia* and *Verrucomicrobiae*, respectively. Meanwhile, the ALE can significantly improve the class of *Betaproteobacteria* (*p* < 0.05). At the level of order classification, we confirmed that the ALE significantly down-regulated Clostridiales (*p* < 0.05) and up-regulated *Verrucomicrobiales* and *Burkholderiales* (*p* < 0.05). Meanwhile, ALE significantly increased the *Actinobacteria*, *Bifidobacteriales* (*p* < 0.05). At the level of family classification, further study on ALE treatment of *Clostridia* showed that there were two significantly declining families, *Eubacteriaceae* and *Ruminococcaceae* (*p* < 0.05), respectively. Interestingly, this class also saw a significant increase in *Clostridiaceae* and *Catabacteriaceae*. At the same time, ALE significantly up-regulated the *Proteobacteria*, *Sutterellaceae* and the *Actinobacteria*, *Bifidobacteriaceae* (*p* < 0.05). At the level of genus classification, three significantly down-regulated genera were identified based on the two down-regulated families of *Firmicutes* (*Butyricicoccus*, *Sporobacter* and *Eubacterium*) and two significantly up-regulated genera of two up-regulated families (*Clostridium_sensu_stricto* and *Catabacter*) (*p* < 0.05). ALE also significantly up-regulated *Bifidobacterium*, *Verrucomicrobiaceae*, *Akkermansia* and *Parasutterella* (*p* < 0.05). Meanwhile, the *Parabacteroides* of *Bacteroidete* was significantly down-regulated by the ALE (*p* < 0.05).

## 4. Discussion

Numerous studies have shown that herbal extracts have positive effects on animal growth. Growth performance is the most intuitive trait index. Pork and chicken are still the main components of meat demand in China. Thus, most studies focus on pig and chicken production. Davila-Ramirez evaluated the effects of mixed plant extracts on different body weight stages of finishing pigs [17]. This study found that between 35 and 120 kg of weight, the mixed extracts could significantly increase the average daily feed intake. Average daily gain also increased significantly from 95 kg. These results showed that the mixed extract could significantly improve the final quality of pigs. However, it is worth noting that this study did not show the effect of extract on improving feed utilization rate. On the contrary, there was a significant decrease in the two stages. Cheng used *Astragalus membranaceus* and *Codonopsis pilosula* extracts to study the growth performance of growing-finishing pigs [18]. It was found that, on the one hand, the final live weight and average daily gain were significantly improved, on the other hand, the feed utilization rate was numerically increased although there was no significant difference. In this study, three different concentrations of ALE were selected, and it was found that the appropriate concentration of ALE could show a trend of increasing the weight of mice. Therefore, we further calculated the feed conversion rate. The results showed that the feed utilization efficiency of the H group was the lowest, and the feed utilization efficiency of the M group and the L group were improved compared with the CK group. The results of this study are similar to those of Zhao’s [19]. He used *Dandelion* root extract and found that it showed a tendency to increase feed utilization while showing no significant difference in feed intake and weight gain for weaned piglets. In summary, feed utilization rate is a key evaluation index, but there is no significant difference in most studies, only the trend of numerical change. This may be attributable to the influence of external environment in animal experiments. The fluctuation of feed intake often leads to a large fluctuation of data, so it is necessary to increase the number of test samples. Meanwhile, plant extracts do have the potential to improve the growth of animals, and are thus worthy of further study.

By measuring the changes of the weight of heart, liver, and spleen, the study showed that the addition of ALE had no significant effect on the changes of weight of all organs. But only the medium concentration of ALE had a significant effect on the increase of the weight of the heart and its relative weight. Wang measured the effects of Chinese herbal medicine extracts on the weight of heart, liver, and spleen, and the results were similar [20]. There was no significant differences in the weight of all organs and their ratio to body weight. However, except liver, they all showed an upward trend. Ding’s study on *Eucommia ulmoides* extracts and Davila-Ramirez’s study on mixed plant extracts did not show significant effects of extracts on various organs [17,21]. From the above and the results of this study, it is easily seen that plant extracts generally have no significant influence on the ratio of organs to body weight. On the one hand, this may be attributable to the small negative impact of natural ingredients on animals. On the other hand, it may be due to the apparent changes not being obvious, and the changes of the internal structure or related functions needs to be further investigated.

The measurement of blood indexes is an effective method to evaluate the nutritional balance of diet and reflect the strength of body resistance. In this study, compared with the CK group, H group significantly increased WBC and GRAN, while M group and L group had no significant effect on blood routine indexes. The increase of these two indexes reflected the possibility of inflammation in the body [22]. Further determination of serum biochemical indicators, liver is the body’s detoxification organ, through the determination that serum ALT and AST activities can reflect the status of the liver [23]. Although there was no significant difference in liver weight among all groups, ALT and AST activities in the H group were significantly higher than those in the other groups. Studies have shown that increased AST and ALT activities suggests adverse effects on the liver. Damage to the liver causes transaminases in liver cells to enter the blood, which raises the levels of ALT and AST in the blood [24]. However, H group had no significant effect on TP, ALB, and GLOB, only showing an increasing trend in numerical value. M group and L group had no significant effect on serum biochemical indices of mice. Therefore, combined with the relevant indexes of blood and serum, it suggests that 300 mg/kg ALE caused damage to the mice and was associated with the risk of inflammation.

Through previous studies, it has been found that the main active substances in the ALE were phenolic acids and flavonoids [16]. Studies have shown that phenols have a low absorption rate in the small intestine, but can play a positive role by regulating intestinal flora [25,26]. Therefore, in order to further determine the effects of ALE on intestinal microflora diversity, the M group (T), with good performance in the above experiments, and the CK group (CON) were selected for 16s rDNA sequencing and bioinformatics analysis. Firstly, through sequencing data filtering and optimization, OTU numbers of CON group and T group were 525 and 503, respectively. Secondly, the dilution curve was analyzed to determine whether the number of samples was sufficient. It could be seen that the curve tended to be flat, and the sequencing depth basically covered all species in the samples. Finally, the RANK curve also showed similar species composition richness and evenness. Based on this, the species richness of samples in this experiment was good under the current sequencing depth, which could be further analyzed. α-diversity analysis was used to comprehensively evaluate whether there were differences between the CON and T groups. Different α diversity indexes can reflect different problems. Sobs, Chao, and Ace reflect the total number of OTU in the samples and are positively correlated with the total OTU number. In this experiment, there were no significant differences between the two groups, and the T group showed a downward trend. Shannon and Simpson reflect the diversity of sample microbial flora. Higher Shannon or lower Simpson indicates higher community diversity. There was no significant difference in this experiment, but the consequences of the two indicators were consistent, and the diversity of the treatment group showed a downward trend. Coverage reflects the coverage of the samples. Despite the fact that the coverage of the T group decreased significantly compared with the CON group, their coverage rates were all greater than 99.9%. Based on the above analysis, it was not difficult to find that extracts have little influence on species differences.

Through Metastats analysis, the composition proportion of different species can be evaluated from different species levels, and species changes brought by ALE can be intuitively reflected in combination with the comparison of key species differences. In this experiment, it can be seen that *Firmicutes* and *Bacteroidetes* were the dominant bacteria in mouse intestine, which conformed to the composition rule of normal mice [27]. Generally speaking, *Firmicutes* are slightly more prevalent than *Bacteroidetes* in the intestinal flora of mice fed a normal diet. Feeding a high fat diet can increase the relative abundance of *Firmicutes* and decrease the relative abundance of *Bacteroidetes* [28]. High fat diets are associated with a range of physical skill disorders, particularly intestinal flora [29]. The disturbance of intestinal flora will lead to the increase of opportunistic pathogens and general pathogens, which will increase the production of endotoxin and cause a series of adverse chain reactions. Meanwhile, the accumulation of toxins can cause chronic inflammation, which can lead to complications [30]. Ge found that the addition of luteolin and metformin alleviated disorders associated with obesity [28]. The adjustment of intestinal microbial composition was the main reason for remission. On the one hand, the abundance of *Bacteroidetes* and *Firmicutes* was restored, and on the other hand, the abundance of *Proteobacteria* was significantly increased. It is worth mentioning that the abundance of *Firmicutes* exceeds that of *Bacteroidetes*. This was consistent with the results of microbial abundance at phylum level in our study. It can be observed that extract treatment can not only adjust the proportion of the two dominant microorganisms, but also improve the abundance of some microorganisms. In addition, the abundance of *Verrucomicrobia* was also increased. The study indicated that the increase of the proportion of *Firmicutes* and *Bacteroidetes* could make the intestinal flora obtain energy more effectively, promote the synthesis of fat and cholesterol, and cause lipid disorders and metabolic diseases [31]. In our study, the ratio of them was significantly decreased in the T group, suggesting that ALE could play a positive role in obesity disorders. Lv found the regulation effect of Ginkgolide B on high-fat mice and drew a similar conclusion. Although *Firmicutes* are the dominant bacteria, the abnormal increase will also bring body disorders [27]. Metastats analysis of the subordination level showed that ALE treatment could significantly improve the abundance of *Akkermansia*, which might be the reason for the increased abundance of *Verrucomicrobia*. Fernando’s study demonstrated the positive regulatory effect of *Akkermansia* on obesity in mice. He found that polyphenols-rich cranberry extract had an alleviating effect on obesity in mice, and its effect on increased *Akkermansia* abundance was determined by metagenomic analysis [26]. Interestingly, regulation of *Akkermansia* by polyphenols was not reported for the first time. Axling found that the regulation of green tea polyphenols on high fat fed mice was also accompanied by an increase in the proportion of *Akkermansia* [32]. Kemperman also proved the up-regulation of *Akkermansia* by compound polyphenols [33]. Combined with the above studies, it is not difficult to see the importance of *Akkermansia* in fat regulation, and polyphenols have huge potential in fat regulation. The ALE in this study are rich in phenolic extracts, which may indicate that the ALE also have a positive effect on the regulation of hyperlipemia.

LEfSe analysis has a more powerful identification function, and evolutionary clade drawn by LEfSe analysis can identify the significant microbiota step by step from the phylum level. Combined with LDA score and Metastats analysis before, it was determined that ALE can regulate the ratio changes of *Bacteroidete* and *Firmicutes* by up-regulating *Parabacteroides* and down-regulating *Butyricicoccus*, *Sporobacter* and *Eubacterium*. This analysis also verified the up-regulation effect of the ALE on *Akkermansia*. It was worth noting that the reason for the increase of *Actinobacteria* discovered by this analysis method was mainly the increase of *Bifidobacterium*. *Bifidobacterium*, an important intestinal beneficial microbe, was also found to have a palliative effect on high-fat mice [34]. In conclusion, we can speculate that ALE can improve the regulation of intestinal lipid by up-regulating intestinal microorganisms *Akkermansia* and *Bifidobacterium*, and thus have a positive effect on intestinal function of mice.

## 5. Conclusions

Addition of 150 mg/kg ALE had positive effects on the growth of mice and improved feed efficiency. Meanwhile, ALE significantly increased the proportion of *Bacteroidetes* and *Firmicutes* and the abundance of *Akkermansia* and *Bifidobacterium*. All results suggest that ALE has a positive regulatory effect on animal growth.

## Figures and Tables

**Table 1:** Identified or tentatively identified compounds in ALE by HPLC-MS under both positive and negative ion mode.

Peak	T_R_ (min)	[M − H]^−^	[M + H]^+^/[M + Na]^+^	Standard Molecular	Molecular Formula	Proposed Compound
1	7.64	353.0868	-/377.0839	354.31	C_16_H_18_O_9_	5-Caffeoylquinic acid
2	10.30	353.0867	-/377.0841	354.31	C_16_H_18_O_9_	3-Caffeoylquinic acid
3	10.80	353.0865	-/377.1787	354.31	C_16_H_18_O_9_	1-Caffeoylquinic acid
4	13.71	563.1399	565.1542/-	564.14	C_26_H_28_O_14_	Apigenin-C-hexaose-C-pentoside
5	18.67	515.1189	-/539.1150	516.12	C_25_H_24_O_12_	3,4-Dicaffeoylquinic acid
6	19.18	515.1191	517.1334/539.1151	516.12	C_25_H_24_O_12_	3,5-Dicaffeoylquinic acid
7	20.37	515.1191	517.1335/539.1152	516.12	C_25_H_24_O_12_	4,5-Dicaffeoylquinic acid
8	23.87	345.0600	347.0761/-	346.06	C_17_H_14_O_8_	Tetrahydroxy-Dimethoxy flavone
9	26.31	359.0756	361.0921/-	360.08	C_18_H_16_O_8_	Centaureidin
10	26.53	329.0649	331.0814/-	330.07	C_17_H_14_O_7_	Jaceosidin
11	29.53	343.0807	345.0969/-	344.08	C_18_H_16_O_7_	Eupatilin

Note: “-” means not detected.

**Table 2:** Nutrient composition of feed.

Items	Content
Moisture (g/kg)	≤100
Crude protein (g/kg)	≥180
Crude fat (g/kg)	≥40
Crude fiber (g/kg)	≤50
Crude ash (g/kg)	≤80
Ca (g/kg)	10–18
P (g/kg)	6–12
Ca:P	1.2:1–1.7:1
Ratio of energy supplied	
Protein (%)	20.6
Fat (%)	12.0
Carbohydrate (%)	67.4
Total energy (kcal/kg)	3530

**Table 3:** Effect of ALE on heart, liver and spleen of mice.

Items	Group				SEM	*p*
	CK	H	M	L		
Average weight (g)	18.48	17.77	19.17	18.22	0.241	0.227
Heart weight (g)	0.097 ^bc^	0.098 ^bc^	0.119 ^a^	0.106 ^b^	0.003	0.007
Liver weight (g)	0.743	0.713	0.760	0.734	0.095	0.391
Spleen weight (g)	0.062	0.061	0.070	0.069	0.016	0.075
H/W (%)	0.525 ^b^	0.551 ^b^	0.623 ^a^	0.582 ^ab^	0.126	0.022
L/W (%)	4.03	4.01	3.97	4.03	0.409	0.949
S/W (%)	0.33	0.34	0.37	0.38	0.080	0.177

Note: CK: 50 μL 0.9% NaCl; H: 50 μL 300 mg/kg ALE; M: 50 μL 150 mg/kg ALE; L: 50 μL 75 mg/kg. Different lowercase letters indicate significant differences.

**Table 4:** Effects of ALE on blood routine in mice.

Items	Group				SEM	*p*
	CON	H	M	L		
WBC (10^9^/L)	2.53 ^b^	2.93 ^a^	2.40 ^b^	2.46 ^b^	0.67	0.001
LYM (10^9^/L)	2.23	2.56	2.13	2.33	0.66	0.089
MON (10^9^/L)	0.10 ^a^	0.00 ^b^	0.00 ^b^	0.03 ^b^	0.14	0.009
GRAN (10^9^/L)	0.5 ^b^	0.73 ^a^	0.33 ^c^	0.5 ^b^	0.47	0.002
RBC (10^12^/L)	10.62	10.36	10.64	10.72	0.18	0.934
HGB (g/L)	143.67	145.67	144.33	144.58	1.88	0.990
HCT (%)	37.77	37.33	37.43	37.70	0.34	0.974
PLT (10^9^/L)	351.00	353.33	356.33	351.67	5.90	0.992

Note: CK: 50 μL 0.9% NaCl; H: 50 μL 300 mg/kg ALE; M: 50 μL 150 mg/kg ALE; L: 50 μL 75 mg/kg. Different lowercase letters indicate significant differences.

**Table 5:** Effects of ALE on serum biochemical indices in mice.

Items	Group				SEM	*p*
	CON	H	M	L		
ALT (IU/L)	26.17 ^b^	45.17 ^a^	27.67 ^b^	26.30 ^b^	2.82	0.011
AST (IU/L)	102.53 ^b^	239.80 ^a^	113.80 ^b^	109.63 ^b^	18.40	0.001
AST/ALT	3.96 ^b^	5.33 ^a^	4.11 ^b^	4.18 ^b^	0.18	0.001
TP (g/L)	68.60	70.47	69.20	66.07	0.80	0.276
ALB (g/L)	36.97	37.73	37.37	35.63	0.43	0.363
GLOB (g/L)	31.53	32.73	31.83	30.43	0.53	0.555
A/G	1.17	1.16	1.19	1.16	0.02	0.955

Note: CK: 50 μL 0.9% NaCl; H: 50 μL 300 mg/kg ALE; M: 50 μL 150 mg/kg ALE; L: 50 μL 75 mg/kg. Different lowercase letters indicate significant differences.

## Data Availability

The data presented in this study are available on request from the corresponding author.
